# 2690. Risk of Invasive Fungal Infections in Patients with Chronic Lymphocytic Leukemia Treated with Bruton Tyrosine Kinase Inhibitors - A Case-Control Propensity Score Match Analysis

**DOI:** 10.1093/ofid/ofad500.2301

**Published:** 2023-11-27

**Authors:** Nelson I Agudelo Higuita, Daniel B Chastain, Brian M Scott, Syeda Sahra, Lilian Vargas Barahona, José Henao Cordero, Alfred L H Lee, Andrés F Henao Martínez

**Affiliations:** University of Oklahoma Health Sciences Center, Oklahoma City, Oklahoma; University of Georgia College of Pharmacy, Albany, Georgia; University of Oklahoma Health Sciences Center, Oklahoma City, Oklahoma; University of Oklahoma Health Sciences Center, Oklahoma City, Oklahoma; University of Colorado Anschutz Medical Campus, Aurora, Colorado; University of Colorado Anschutz Medical Campus, Aurora, Colorado; Prince of Wales Hospital, Sha Tin, Not Applicable, Hong Kong; University of Colorado Anschutz Medical Campus, Aurora, Colorado

## Abstract

**Background:**

Invasive fungal infections (IFIs) have been reported with increasing frequency in patients with chronic lymphocytic leukemia (CLL) treated with Bruton tyrosine kinase inhibitors (BTKi). High-quality real world data examining the frequency of IFIs and identifying risk factors to guide preventive measures in this patient population is lacking. We characterized the risk of IFIs among patient with CLL on BTKi one year after diagnosis using a global health research network.

**Methods:**

We used TrinetX to identify patients with CLL based on diagnosis codes and stratified based on treatment with BTKi. Patients from each group were matched based on demographics, comorbidities, and medications to analyze the frequency of IFIs and outcomes at one year after CLL diagnosis.

**Results:**

A total of 17,624 patients with CLL underwent propensity score matching. Approximately 82% of patients were white, 64% were male with a mean age of 69.5 years. Use of systemic corticosteroids, anti-CD20 antibodies, voriconazole, and fluconazole was higher in patients treated with BTKi (p < 0.001 for each drug). TMP/SMX administration was slightly higher in the BTKi group (18% vs. 17%, p = 0.01). At one year after CLL diagnosis, hospitalization was similar among groups (12.2% vs. 11.15%, p = 0.07), while mortality was lower in patients with CLL on a BTKi (9.9% vs. 12.3%, p < 0.001). Invasive candidiasis was less common (1.6% vs. 2.1%, p = 0.01) while pneumocystis was more common (0.3% vs. 0.1%, p = 0.007; risk ratio: 2.6, 95% CI: 1.3-5.4) in patients taking BTKi one year following diagnosis. The proportion of patients with invasive aspergillosis (0.1% vs. 0.2%, p = 0.143) or cryptococcosis (0.2% vs. 0.1%, p = 0.238) was similar between groups.

**Conclusion:**

The overall frequency of IFIs was very low in patients with CLL on BTKi despite a higher frequency of steroid administration and only significant for PJP, albeit at a low annual risk of 0.3%. We calculated 500 patients needed prophylaxis to prevent one episode of PJP in patients with CLL on BTKi. This finding does not support routine PJP primary prophylaxis, considering the historical PJP risk of greater than 6.2% per person-year as the recommended threshold for primary prophylaxis in non-HIV immunocompromised patients.

**Disclosures:**

**All Authors**: No reported disclosures

